# Unveiling a potential threat to forest ecosystems: molecular diagnosis of *Alliaria petiolata*, a newly introduced alien plant in Korea

**DOI:** 10.3389/fpls.2024.1395676

**Published:** 2024-07-01

**Authors:** Tae-Young Choi, Dong Chan Son, Ami Oh, Soo-Rang Lee

**Affiliations:** ^1^ Department of Biology Education, College of Education, Chosun University, Gwangju, Republic of Korea; ^2^ Division of Forest Biodiversity and Herbarium, Korea National Arboretum, Pocheon, Republic of Korea

**Keywords:** invasive species, polyploidy, RAD-seq, chromosome number, admixture, *Alliaria petiolata*

## Abstract

Identifying stages of a species invasion in a new habitat (i.e., colonization, establishment, and landscape spread) and their primary determinants in biological invasion warrants attention, as it provides vital insights for preventing non-native species from becoming pervasive invaders. However, delineating invasion stages and their associated factors can pose significant challenges due to the ambiguous distinctions between these stages. *Alliaria petiolata*, one of the most noxious weeds in woodland habitats, has recently been introduced to Korea and observed in a few distant locations. Although the plant’s spread has been relatively slow thus far, rapid spread is highly likely in the future, given the high invasive potential reported elsewhere. We indirectly diagnose the current status of *A. petiolata* invasion in Korea through the assessment of genetic diversity and phylogenetic inferences using genome-wide molecular markers and cytological data. We analyzed 86 individual samples collected from two native and six introduced populations, employing 1,172 SNPs. Our analysis estimated within- and among-population genetic diversity and included two clustering analyses. Furthermore, we investigated potential gene flow and reticulation events among the sampled populations. Our data unraveled that Korean garlic mustard exhibits a hexaploid ploidy level with two distinct chromosome numbers, 2n = 36 and 42. The extent of genetic diversity measured in Korean populations was comparable to that of native populations. Using genome-wide SNP data, we identified three distinct clusters with minor gene flow, while failing to detect indications of reticulation among Korean populations. Based on the multifaceted analyses, our study provides valuable insights into the colonization process and stressed the importance of closely monitoring *A. petiolata* populations in Korea.

## 1 Introduction

Biological invasion poses great threats to biodiversity, ecosystem integrity, human health, and the economy worldwide (Usher, 1988; Westbrooks, 1998; Nuzzo, 1999; Kolar and Lodge, 2001; Hulme, 2006; Cuthbert et al., 2022; Fantle-Lepczyk et al., 2022). It is a complex process consisting of four consecutive stages (transport, colonization, establishment, and spread) that vary spatially and temporally (Theoharides and Dukes, 2007). On each stage, a series of environmental filters and eco-evolutionary factors act as critical determinants of invasion success (Simberloff, 2009; Dlugosch et al., 2015; Blackburn et al., 2019; Sherpa and Després, 2021; Gioria et al., 2023). Identifying the stages, thus, can provide valuable insights into the primary determinants during the species colonization. Detecting early stages of invasion before the final stage (spread) can grant us critical information to prevent invaders from further spreading. However, it can be very challenging as there is often no clear distinction between the stages (Theoharides and Dukes, 2007). Detecting the invasion stages can be further complicated if one of the stages is delayed, leading to a lag phase.

After transport, propagule pressure, i.e. the number of introduced individuals or sources during biological invasion (Kolar and Lodge, 2001), significantly affects the success of colonization stage (Lockwood et al., 2005; Theoharides and Dukes, 2007). High propagule pressure can greatly contribute to ameliorating reductions in genetic diversity, a significant barrier to biological invasion, particularly in the early stages. Colonizers may possess well-maintained genetic variation to cope with abiotic filters, such as climate and resource availability, which are determinants of colonization success (Sakai et al., 2001; Tilman, 2004; Leishman and Thomson, 2005; Theoharides and Dukes, 2007). Self-compatibility of an invader can also be advantageous in colonization since it facilitates finding mates and getting established in novel condition (Baker, 1955). In the third stage, “establishment”, biotic filters are the most critical barrier to the invasion success. Invaders often face competition with native plants and other invaders during the stage. Along with allelopathic agents, polyploidy plays important roles in the success of the stage through mechanisms such as heterosis, hybrid vigour and prevention from the effects of deleterious recessive mutations (Te Beest et al., 2012; Suda et al., 2015; Mairal et al., 2023).

Successful invaders may ultimately spread into varying landscapes, expanding their distribution (Theoharides and Dukes, 2007). During the spread, the invader’s dispersal ability and habitat connectivity are major determinants (Theoharides and Dukes, 2007). In addition, a lag phase can often be observed between establishment and spread, but the stage is extremely hard to detect due to the lack of clear and observable distinctions from establishment (Theoharides and Dukes, 2007). Previous studies utilizing simulation and modeling have documented the significant challenge in predicting the lag phase, which is crucial for weed management (Crooks, 2005; Coutts et al., 2018). This stage often reflects a low level of genetic variation in the invader population or the time required for the population to reach a size conducive to spreading (Sakai et al., 2001; Barney, 2006). The stage also can indicate a lack of suitable habitat for the invader (Pyšek and Hulme, 2005). Therefore, the lag phase deserves more attention in the study of invasion. A study on weeds in New Zealand revealed that some lag phases, e.g. *Cytisus scoparius* and *Sambucus nigra* can extend nearly 100 years, while another study on exotic plants of USA documented the lag times ranging from 3 to 140 years (Aikio et al., 2010; Larkin, 2012; Coutts et al., 2018).

Garlic Mustard, *Alliaria petiolata* (Bieb.) Cavara & Grande (Brassicaceae), is a biennial herb, occasionally being a perennial, native to Europe and Southwestern Asia (Welk et al., 2002; Prati and Bossdorf, 2004; Cho and Kim, 2012). In the first year, plants germinate and form basal rosettes, which persist through the winter, while in the next year, they flower and produce seeds (Cavers et al., 1979). *Alliaria petiolata* adopts both cross- and self-fertilization, with selfing being the predominant breeding system (Durka et al., 2005; Mullarkey et al., 2013). The plant primarily relies on insect-mediated pollination, by flies and bees (Cruden et al., 1996). Notably, according to Baker’s law, self-compatibility contributes to the success of invasion, explaining the prevalence of self-compatible traits among weeds (Baker, 1955). *Alliaria petiolata* reproduces and disperses exclusively by seeds (Nuzzo, 1999), with individual plants capable of producing hundreds of seeds (Nuzzo, 1993). Seed dispersal occurs naturally up to distances of 100 meters or more, facilitated by various animals, including humans (Lhotská, 1975; Cavers et al., 1979; Nuzzo, 2000). *Alliaria petiolata* was likely introduced to North America in the 19th century, either for medicinal and culinary purposes or inadvertently as a byproduct (Grieve, 1959; Nuzzo, 1993; Meekins and McCarthy, 1999). For decades, the plant has rapidly invaded deciduous forests in the northern United States and southern Canada (Meekins and McCarthy, 1999; Nuzzo, 1999). As one of the most notorious invaders of woodland habitats, *A. petiolata* has aggressively displaced native plants in introduced regions, consequently prompting the implementation of biological control programs (Blossey et al., 2001). Previous studies have highlighted competitive ability and allelopathy as the primary mechanisms driving the successful invasion of this species (Kelley and Anderson, 1990; Meekins and McCarthy, 1999). *Alliaria petiolata* can adversely affect soil microbiota and native plants by producing secondary compounds such as glucosinolates and their degradation products (Vaughn and Berhow, 1999; Cipollini et al., 2012; Cipollini and Cipollini, 2016). High seed production may also be attributable to the invasive success of *A. petiolata* (Anderson et al., 1996).

Notably, both diploids (2n = 14) and hexaploids (2n = 36, 42) have been identified within the species (Frisch and Møller, 2012; Alabi et al., 2021). Diploids are reported from Western Asia, while hexaploids are found in Central/Western Europe and North America (Weiss-Schneeweiss and Schneeweiss, 2003; Esmailbegi et al., 2018; Alabi et al., 2021). Based on the observed chromosome numbers in the species, a haploid chromosome number of n = 7 has been assumed. Interestingly, a small number of accessions with 2n = 36 occurred in the Netherlands and Sweden, indicating the existence of n=6 in the species (Gadella and Kliphuis, 1963, 1966; Frisch and Møller, 2012). In a previous phylogenetic study, *A. petiolata* populations from Asia, Europe and North America formed two distinct clades, representing the existence of two different ploidy levels within the species (Esmailbegi et al., 2018). To date, the origin of the hexaploid cytotype, including whether hexaploid in the species is autopolyploid or allopolyploid, remains largely unexplored (Frisch and Møller, 2012; Bayat et al., 2021). However, recently, allopolyploid origin of hexaploid *A. petiolata* was suggested based on the reconstruction of genome structure (Bayat et al., 2021).

**Figure 1:**
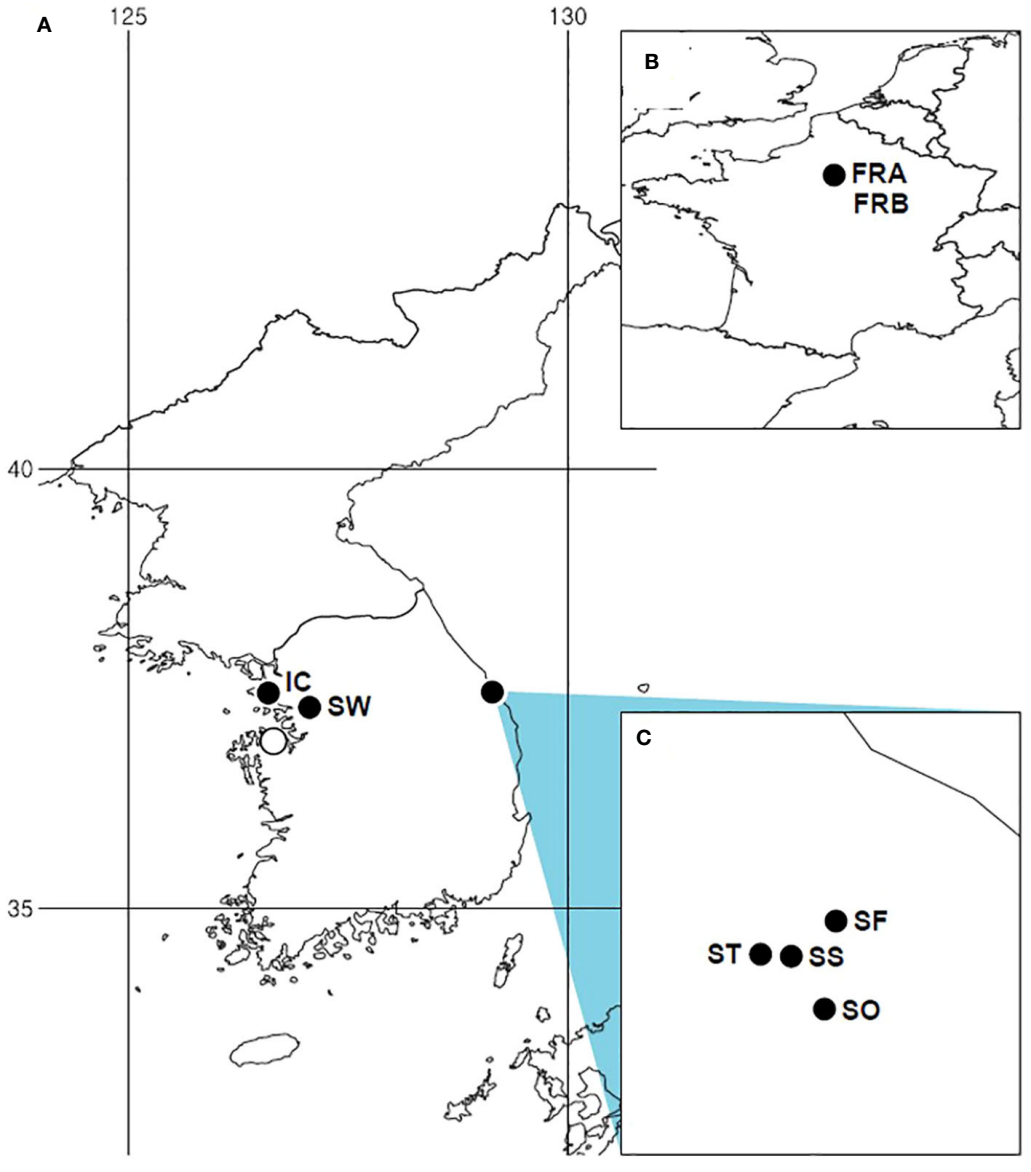
Distribution of *Alliaria petiolata* sampling sites and occurrences in the introduced region (South Korea). **(A)** Distribution of *Alliaria petiolata* in Korea. **(B)** Sampled populations in France, the native region. **(C)** Four populations sampled in Gangwon-do. All filled circles indicate sampling sites. Population acronyms are listed in **Table 1**.

In South Korea, *A. petiolata* was initially spotted in Samcheok (Gangwon-do), the eastern coastal region, during a floristic survey in 2012 (Cho and Kim, 2012). A small number of populations in this region are situated along forest edges, easily accessible to people as they spread along roadsides. Subsequently, the species was detected in Incheon, Suwon, and Dangjin, the westernmost areas of South Korea (**Figure 1**). In particular, the population in Dangjin was only recently observed, in 2022. The invasion of the species in Korea is not yet severe, as it has only colonized a few restricted areas with a limited number of populations. Given the absence of rapid population growth or range expansion, the current status of invasion can be considered to be in a lag phase, although further investigations are needed to make a more precise determination of the invasion stage. With the aforementioned backgrounds, the specific objectives of our research were: (1) to reestablish the identity of Korean *A. petiolata*, (2) to assess and compare the genetic structure, genetic diversity, and genetic divergence between the native and introduced populations, (3) to infer the stage of *A. petiolata* invasion in Korea. To address these goals, we used a large number of molecular markers across the whole genome and collected samples from the majority of available populations in Korea. Population-level genetic studies offer valuable insights for understanding, reconstructing, and managing invasions (Ficetola et al., 2008; Stewart et al., 2009; Estoup and Guillemaud, 2010; Cristescu, 2015; Lee and Son, 2022).

## 2 Materials and methods

### 2.1 Sample collection and chromosome counting

We collected 108 samples in the summer of 2022 from 6 populations distributed in Korea (**Figure 1**). Nearly all *A. petiolata* populations in Korea reported at the time of sampling were included. We, additionally, collected 20 samples originated from two native populations (**Table 1**). We carefully chose the individual samples to avoid collecting multiple samples of a single plant by distancing more than 10m among the sampled plants. Young leaf tissues were picked and preserved at room temperature in a sealed plastic Ziplock bag with silica desiccant.

**Table 1:** Summary of collection sites and within-population genetic diversity estimates across 8 populations of *Alliaria petiolata*.

Location	Population acronym	GPS		N	Ne	Ne [SE]	NBOT	NBOT [SE]	Na Rare	Na Rare [SE]	He	He [SE]	Ho	Ho [SE]
		X	Y											
Uji-dong, Samcheok-si, Gangwon-do	SF	37.46138	129.15583	14	21	0.599	6	0.056	1.44	0.014	0.212	0.007	0.410	0.014
Uji-dong, Samcheok-si, Gangwon-do	SO	37.44793	129.15407	12	31	0.601	5	0.05	1.4	0.014	0.197	0.007	0.384	0.014
Uji-dong, Samcheok-si, Gangwon-do	SS	37.456	129.149	12	14	0.409	5	0.056	1.4	0.014	0.197	0.007	0.386	0.014
Uji-dong, Samcheok-si, Gangwon-do	ST	37.4563	129.1443	14	16	0.288	6	0.025	1.4	0.014	0.197	0.007	0.387	0.014
Ha-dong, Yeongtong-gu, Suwon-si, Gyeonggi-do	SW	37.282029	127.065605	15	23	0.599	11	0.226	1.42	0.014	0.206	0.007	0.405	0.014
Hang-dong, Jung-gu, Incheon	IC	37.445	126.59805	8	53	1.76	11	0.226	1.41	0.014	0.198	0.007	0.357	0.013
Jardin botanical garden, Paris, France	FRA	48.844667	2.361972	5	–	–	–	–	1.58	0.0145	0.273	0.007	0.388	0.014
Lac Daumesnil, Paris, France	FRB	48.829944	2.4125	4	–	–	–	–	1.44	0.0145	0.204	0.007	0.406	0.014

N, sample size; Lat and Lon, geographic coordinates in decimal degrees; Ne, effective population size; NBOT, effective size of bottlenecked population; Na_Rare, mean number of alleles adjusted by population size across 1,172 SNPs; He, mean expected heterozygosity across 1,172 SNPs; Ho, mean observed heterozygosity across 1,172 SNPs and SE, standard error.

Garlic mustards can both be diploids (2n=14) and hexaploids (2n=36, 42; Frisch and Møller, 2012; Alabi et al., 2021). Thus, we determined the ploidy level of the plant with chromosome counting. We collected a whole plant including roots from 6 Korean populations and cultivated the roots for 2 weeks in a tap water. The chromosome counting was carried out following Dematteis and Fernández (1998) with well-developing roots. We prepared slides and analyzed at least 3 metaphase cells that were showing well-spread chromosomes for chromosome counting. Chromosome numbers were determined under Leica DM3000 LED microscope (Leica Microsystems, Wetzlar, Germany) and photographed by Dhyana 400DC (Tucsen Photonics sCMOS, Fuzhou, China).

### 2.2 DNA extraction, library preparation and genotyping

Total genomic DNA was extracted from the preserved tissues using the DNeasy Plant Mini Kit (Qiagen, Hilden, Germany), using the manufacturer’s protocol. The isolated genomic DNA was then checked for quality by visualizing in a 1% agarose gel. Quantification was assessed by Qubit 4 Fluorometer (Thermo Fisher Scientific, MA, USA) and stored at -20°C.

We employed 3RAD (Bayona-Vásquez et al., 2019) approach to genotype the collected samples, which improves the adapter ligation efficiency by employing a third restriction enzyme to cleave adapter dimers. We prepared the library following Bayona-Vásquez et al. (2019) with a few adjustments. Three enzymes, EcoRI-HF (common cut), XbaI (rare cut), and third enzyme NheI for the dimer cleaving (all enzymes from Thermo Fisher Scientific) were applied to digest the genomic DNAs. After a series of adapter ligation, clean-ups and amplifications, we targeted and selectively collected 500-bp fragments (+/− 10%) using Pippin Prep (Sage Science, MA, USA). The library preparation was completed with a final amplification. We then evaluated the prepared library on Agilent 2100 Bioanalyzer (Agilent Technologies, Santa Clara, CA, USA) and sent it to Macrogen Inc (Korea). The final library was sequenced on Illumina HiSeq X-10 platform with 2 × 150 paired-end sequencing.

**Figure 2:**
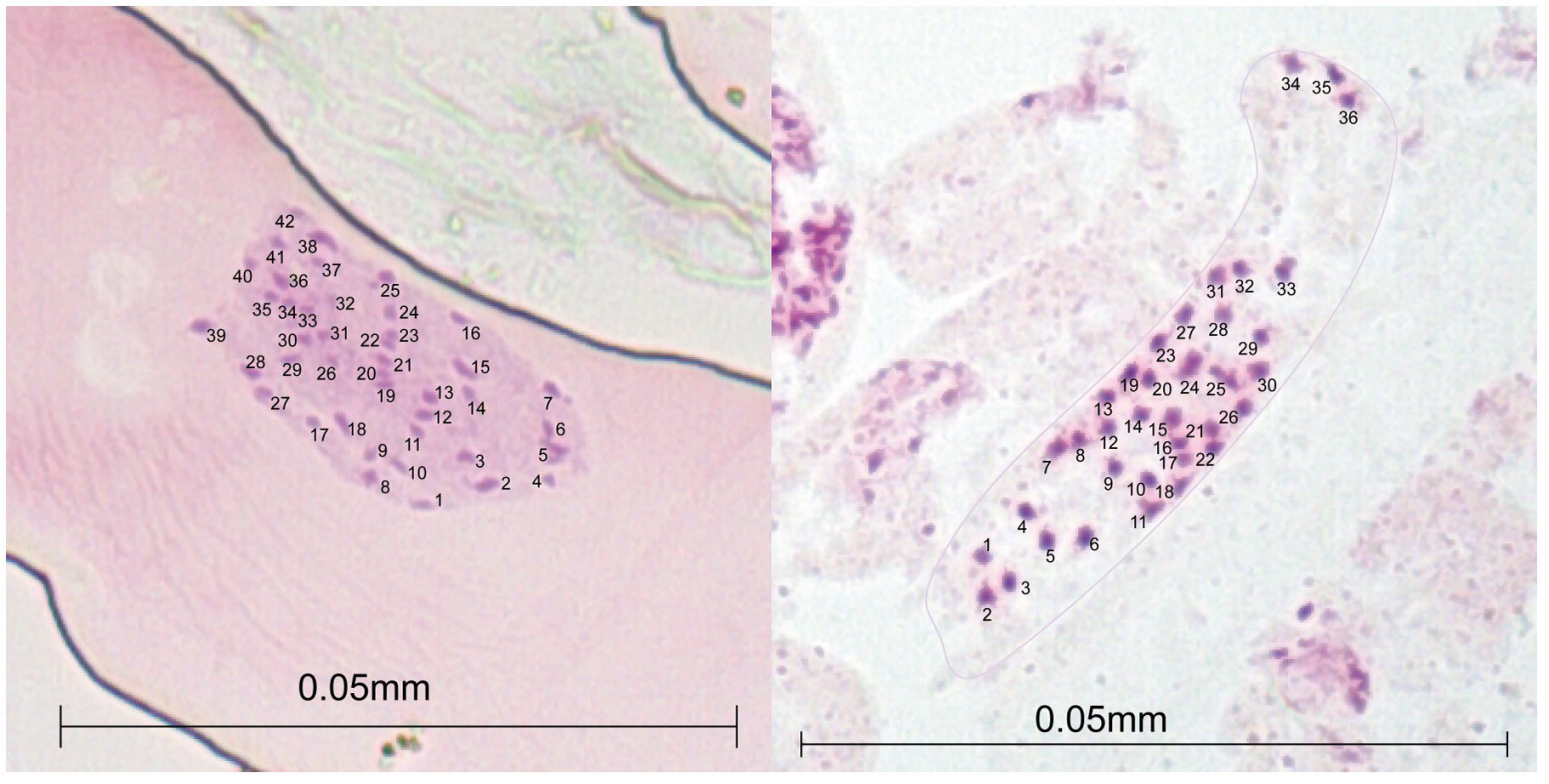
Acetocarmine-stained mitotic metaphase chromosome counts of root meristem in two *Alliaria petiolata* populations (SS and SF; see population details in **Table 1**) of Korea showing two hexaploid karyotypes (2n = 36 and 42).

The raw sequence data were first demultiplexed and trimmed using -t 141 in Stacks v. 2.41 (Rochette et al., 2019). We used process_radtags function to filter out bad quality reads with high error rate (threshold Phred score 10 and sliding window size 0.15). The filtered reads were then mapped to a reference genome (GCA_020283515.1) using Bowtie v. 2.2.3 with MAPQ < 30 (Langmead and Salzberg, 2012). Given the multiple ploidy levels (diploid and hexaploid) observed within the species (X= 6 and 7; 2n= 36 and 42; **Figure 2**), we assembled the reads and called SNPs on ipyrad v.0.9.93 (**Supplementary Information 1**; Eaton and Overcast, 2020). Initially, we assembled the catalogs with a clust threshold of 90% and a minimum depth of 6 reads for base calling. Additionally, we implemented a threshold allowing a maximum of 6 alleles per site to accommodate the potential hexaploid sampled during the final cluster filtering.

To ensure independence among called SNPs, we only included the first SNP per locus by using u option during step 7 of ipyrad pipeline. SNP loci significantly departing from Hardy-Weinberg Equilibrium (HWE, P < 10e-6; Li, 2011; Hess et al., 2012) were also filtered to exclude loci with extreme heterozygosity that were likely resulting from assembly errors in Plink v. 1.9 (Purcell et al., 2007). We finalized our data to 1,172 SNPs for 86 genotypes by additionally purging genotypes with more than 30% missing calls and SNP loci with minor allele frequency of ≤ 0.05 using Tassel 5.0 (Glaubitz et al., 2014).

### 2.3 Data analysis

Three genetic diversity parameters, expected heterozygosity (He), observed heterozygosity (Ho) and number of alleles (Na) were estimated in GENALEX v. 6.5 (Peakall and Smouse, 2012). Due to unequal sample sizes across regional populations, Na values were adjusted using rarefaction curves (**Table 1**; Kalinowski, 2004) in HP-Rare (Kalinowski, 2005). We computed pairwise population differentiation (FST) among 8 populations using 1,000 permutations to estimate the statistical support in Arlequin v. 3.5 (Excoffier and Lischer, 2010). A Mantel test was conducted to examine a significant isolation by a geographic distance. For the Mantel test, we used log-transformed Euclidean distances as predictors and linearized FST [FST =FST/(1- FST)] values as regressors in GENALEX (Rousset, 1997).

We utilized a simulation-based approach implemented in FASTSIMCOAL2 (Excoffier et al., 2013) to calculate the effective population size (Ne) under various demographic scenarios for 6 populations sampled in the introduced region (Korea). To reduce model complexity, we examined three simple models focusing on a single population. These models are detailed as follows: 1) null model with constant population size, 2) population bottleneck model and 3) population bottlenecks and rebound model. For each demographic model, we adopted a mutation rate of 7 × 10^-9 estimated from *Arabidopsis thaliana* (Krasovec et al., 2018). We computed folded site frequency spectra (SFS) for SNP loci separately for the seven local populations to mitigate the effect of missing sequence data. In the composite likelihood computation, we conducted 400,000 simulations and 80 ECM (Expectation Conditional Maximization) cycles. This process was repeated 100 times for each demographic model with a stopping criterion of 0.001 (Excoffier and Foll, 2011; Excoffier et al., 2013). We identified the optimal run for each demographic model based on maximum likelihood and subsequently calculated the AIC scores. The best demographic model for each population was selected based on the AIC values.

We determined assignment patterns among 8 populations using two differing approaches, Principal coordinate analysis (PCoA) and Bayesian model-based assignment test. PCoA was performed based on Nei’s genetic distance calculated from 86 genotypes in GENALEX. The number of randomly mating clusters (K) was determined using STRUCTURE v. 2.3.4 (Pritchard et al., 2000) as implemented in ipyrad. We conducted all STRUCTURE analyses using an admixture model with a burn-in period of 100,000 steps, followed by 1,000,000 Markov Chain Monte Carlo (MCMC) iterations. The allele frequency model was set to the independent model, which is widely applied and assumes distinct allele frequencies for different populations. Values of K ranging from 1 to 8 were tested, with each K run repeated 10 times for robustness and consistency. We employed Structure Harvester version 0.6.93 (Earl and VonHoldt, 2012) to determine the optimal K values using both the delta K method (Evanno et al., 2005) and the approach outlined in Pritchard et al. (2000). The latter selects the K value at the point where the natural logarithm of the likelihood of the data given K (lnK) plateaus. In cases where the optimal K values conflicted between the two methods, we prioritized the former method, unless delta K values exceeded 2. This decision was based on findings suggesting that the delta K method tends to underestimate the optimal value of K (Janes et al., 2017). To summarize and visualize the ancestry coefficients inferred for the optimal K, we used clumpp v. 1.1.2 (Jakobsson and Rosenberg, 2007) as implemented in ipyrad.

**Figure 3:**
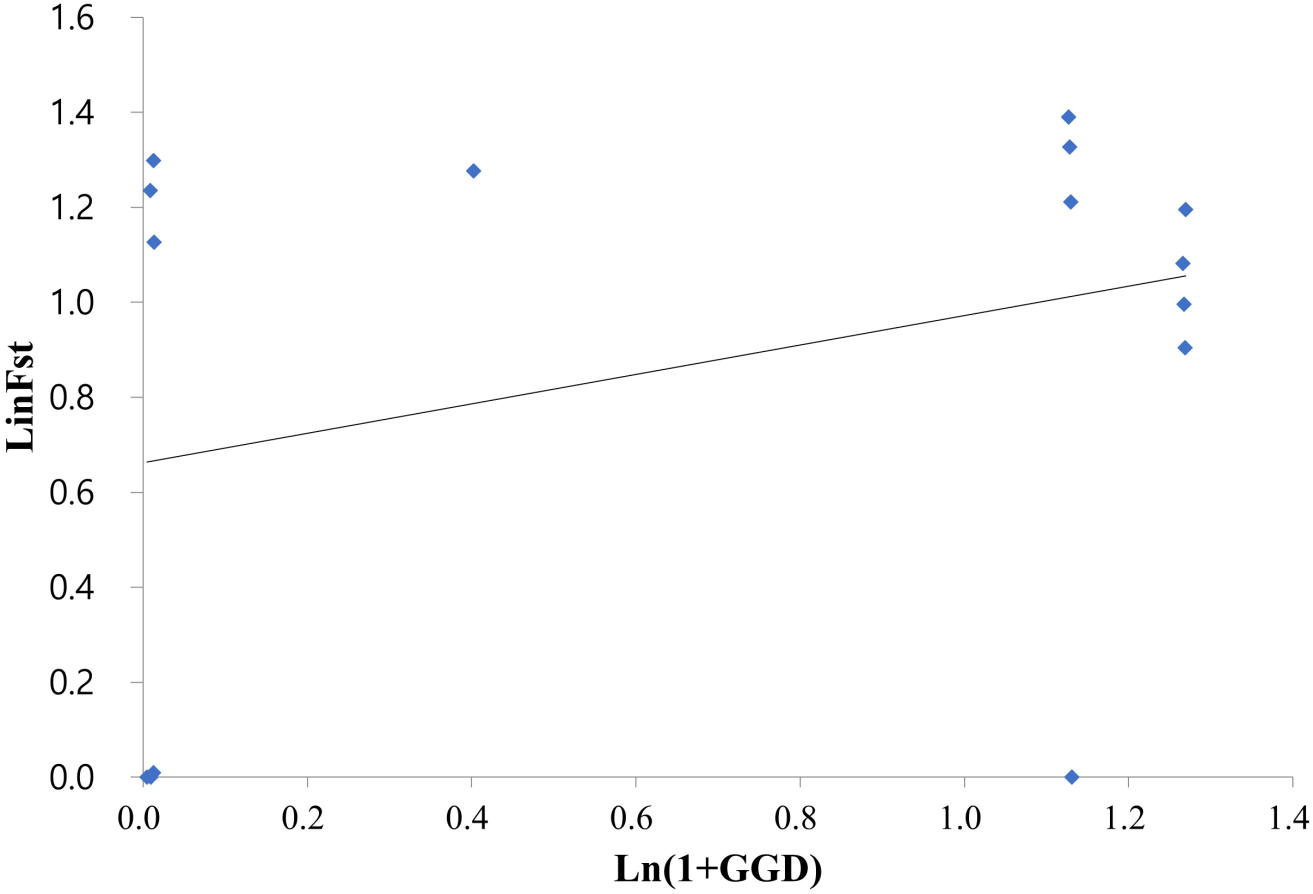
Relationship between the genetic and geographic distances among the six Korean *Alliaria petiolata* populations assessed by a Mantel test (r = 0.33, p < 0.05). The test employed the logarithm of Euclidean distance (km) and Slatkin’s linearized FST (FST/(1 − FST)) values.

**Figure 4:**
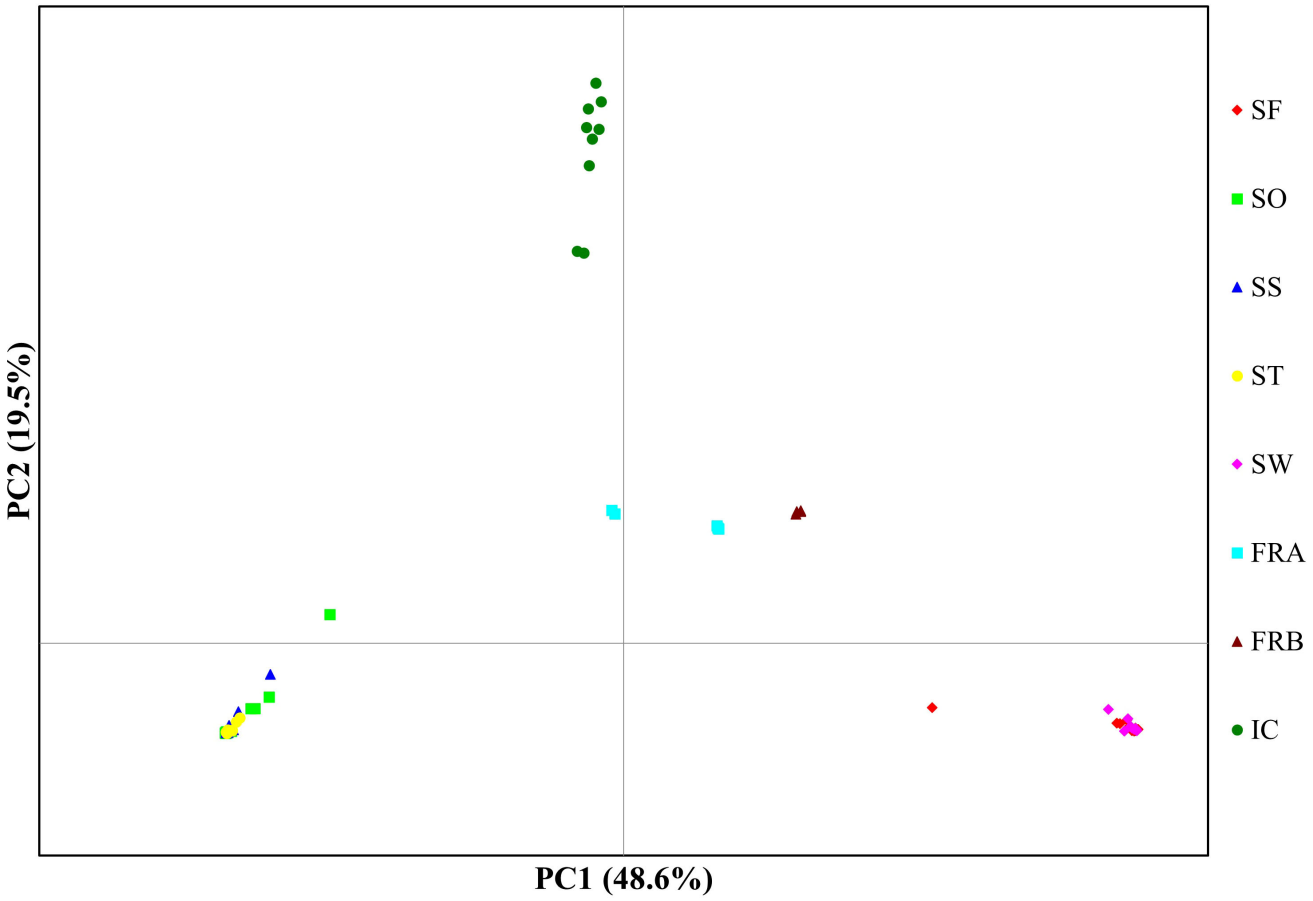
Plot for principal components analysis of 86 *Alliaria petiolata* genotypes from 8 populations. The first two PC axis were plotted. Refer **Table 1** for acronyms of population locations and sample sizes.

We reconstructed a phylogenetic network using NeighborNet algorithm implemented in SplitsTree v. 4.17.1 (Huson and Bryant, 2006) to examine potential reticulation events among the 86 genotypes. We used the 1,172 loci to estimate genetic distance among the 86 genotypes. Genetic distance was estimated with K2P model and applied to infer the phylogenetic network. We also explored gene flow among the sampled populations in TreeMix v. 1.13 (Pickrell and Pritchard, 2012). The 1,172 SNP data were converted into the TreeMix input file using populations function in Stacks program. We rooted trees with the genotypes of IC population (-root) since those genotypes exhibited the most distant genetic relations with the rest of the samples (**Table 2**; **Figures 3**, **4**). To estimate the covariance matrix, 500 bootstrap replicates were generated with a SNP block size of 100 (-K) per migration edge. Gene flow among populations was tested using 0 ~ 5 migration edges (m = 0 ~ 5). We employed R package OptM (Fitak, 2021) to find the optimal number of migration events.

**Table 2:** Mean pairwise F_ST_ values estimated from 1,172 SNPs among 8 *Alliaria petiolata* populations in Korea.

	SF	SO	SS	ST	SW	IC	FRA	FRB
**SF**	0.000							
**SO**	0.411	0.000						
**SS**	0.412	0.003	0.000					
**ST**	0.411	0.004	0.002	0.000				
**SW**	0.005	0.417	0.418	0.417	0.000			
**IC**	0.422	0.395	0.396	0.397	0.441	0.000		
**FRA**	0.277	0.283	0.283	0.283	0.289	0.301	0.000	
**FRB**	0.319	0.406	0.406	0.407	0.342	0.402	0.231	0.000

All values except for the values in shaded area were statistically significant at P < 0.01 level.

## 3 Results

We initially tried to count the chromosome numbers for at least one sample from all six populations collected from Korea. However, due to difficulties in root sample preparations, we failed to successfully count the chromosome numbers for four of the six samples used in the chromosome counting. The chromosome numbers for samples from SF and SS populations (see **Table 1** for population acronyms and the detailed location information) were only appropriately counted. The chromosome number of SS population was 36, whereas that of SF population was 42, indicating a clear difference between the two populations in the basic chromosome number (**Figure 2**).

The library produced 41.9 Gbp with 279 million raw reads (270,820-6,726,414 reads per sample). The average GC content was 43.9% differing from the reference genome (37%, GCA_020283515.1). The average mapping rates differed across populations ranging from 49.8 (SF) to 56.1 (FRA). We initially isolated 153,272 SNP loci but, after a series of filtering processes, 1,172 SNPs with low missing rates (< 10%) remained for the downstream analyses. The final transition/transversion rate of SNP matrix was 1.617. Overall, the genetic diversity of *A. petiolata* was consistent across 8 populations. The number of alleles rarefied were between 1.40 to 1.58 (mean Na_Rare = 1.44), and the expected heterozygosity (He) was ranged from 0.197 to 0.273 (mean He = 0.211; **Table 1**). Notably, we found much higher variation in observed heterozygosity (mean Ho = 0.4) comparing to He (**Table 1**). As opposed to our expectation, no significant difference in genetic diversity measures was observed between the native and the introduced populations.

On average, populations highly diverged from each other (average F_ST_ = 0.314). Pairwise F_ST_ largely varied across populations ranging from near zero (SW/SF, SS/ST, SS/SO and SO/ST pairs) to 0.441 (SW/IC pair; **Table 2**). All F_ST_ values were statistically significant except for the four population pairs with extremely low F_ST_. Notably genetic divergence among some local population pairs were comparable or even greater than the one between the introduced and the native populations (**Table 2**). For instance, IC population largely diverged from the remaining five domestic populations in Korea (F_ST_ = 0.301 - 0.422). The values were, in fact, comparable or much greater than the F_ST_ estimates for the population pairs of the native populations (FRA or FRB) with the six Korean populations (F_ST_ = 0.28 – 0.41; **Table 2**). According to the Mantel result, the genetic divergences among 6 domestic populations were not significantly related to the geographic distances (**Figure 3**; r = 0.33, P > 0.5).

We further assessed the Ne for six populations sampled in Korea using FASTSIMCOAL with three demographic models. The model that best explained our SNP data for all six populations was the population bottlenecks and rebound model. According to the model, the six populations in Korea initially underwent population bottlenecks followed by population growth. The estimated Ne at the bottleneck point ranged from 5 (SO) to 11 (SW, IC), while the Ne of the current populations, rebounding from the decline, ranged from 14 (SS) to 53 (IC; see **Table 1**).

**Figure 5:**
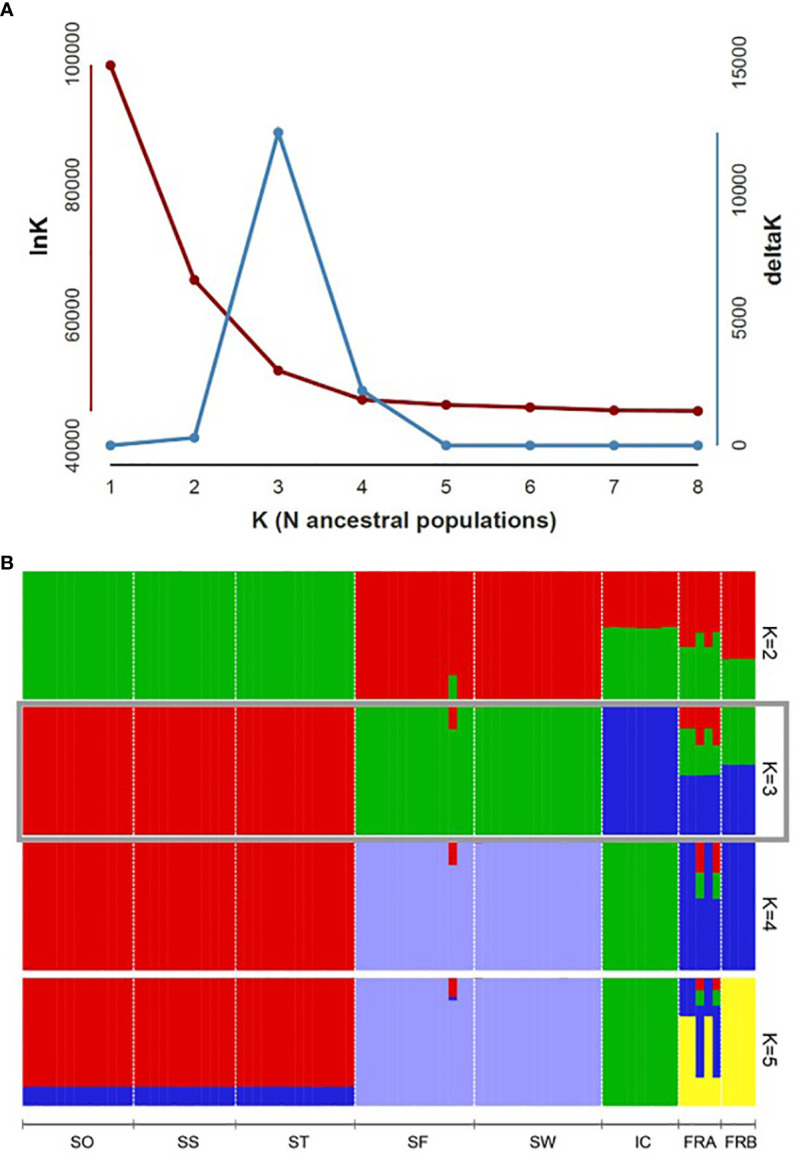
Population genomic structure of 8 *Alliaria petiolata* populations. **(A)** Plot of delta K estimated, following Evanno et al. (2005), to determine the optimal K numbers. **(B)** Bar plot visualizes group assignments for 86 genotypes of the optimal K (K = 3). Populations are separated by the vertical black lines. Refer **Table 1** for acronyms of population locations and sample sizes.

PCoA plot with the axes PC1 and PC2, where PC1 explains about 49% of the total variance and PC2 about 20%, identified four clusters (**Figure 4**). PC1 separated the 8 populations into 4 groups, while PC2 split the populations into two groups (**Figure 4**). The populations SO, SS, and ST in Samcheok clustered together on the left side of the plot, and were separated from the second cluster composed of SF (Samcheok) and SW (Suwon), on the right side (**Figure 4**). Along PC1 axis, in the middle of these two clusters, IC population from Incheon was located (**Figure 4**). The two native populations from France were clustered together in the middle of the PC plot (**Figure 4**). STRUCTURE results exhibited similar clustering pattern. The best K determined based on delta K was K=3 (**Figure 5A**). When K=3, all genotypes from the populations SO, SS, and ST (Samcheok) were assigned to cluster 1 represented in red, whereas all genotypes from SF (Samcheok) and SW (Suwon) were assigned to cluster 2 in green except for one genotype in SF (**Figure 5B**). The IC population was assigned to another cluster 3 in red (**Figure 5B**). Native populations showed admixed patterns among all three clusters (**Figure 5B**).

**Figure 6:**
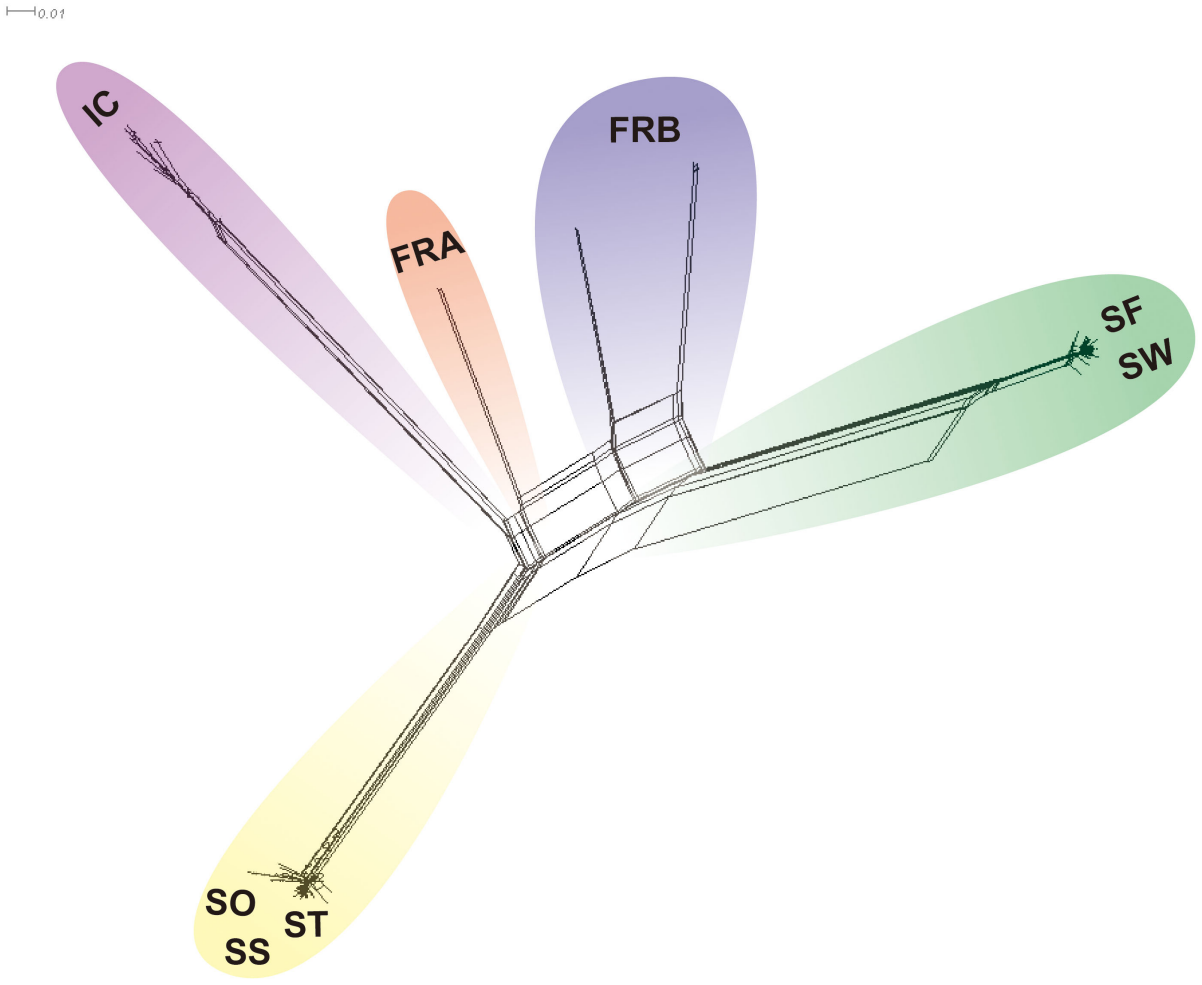
NeighbourNet tree illustrating the phylogenetic relationship of 86 *Alliaria petiolata* samples, based on uncorrected-p distance. Colored shadings highlight clades with population samples assigned to each clade.

**Figure 7:**
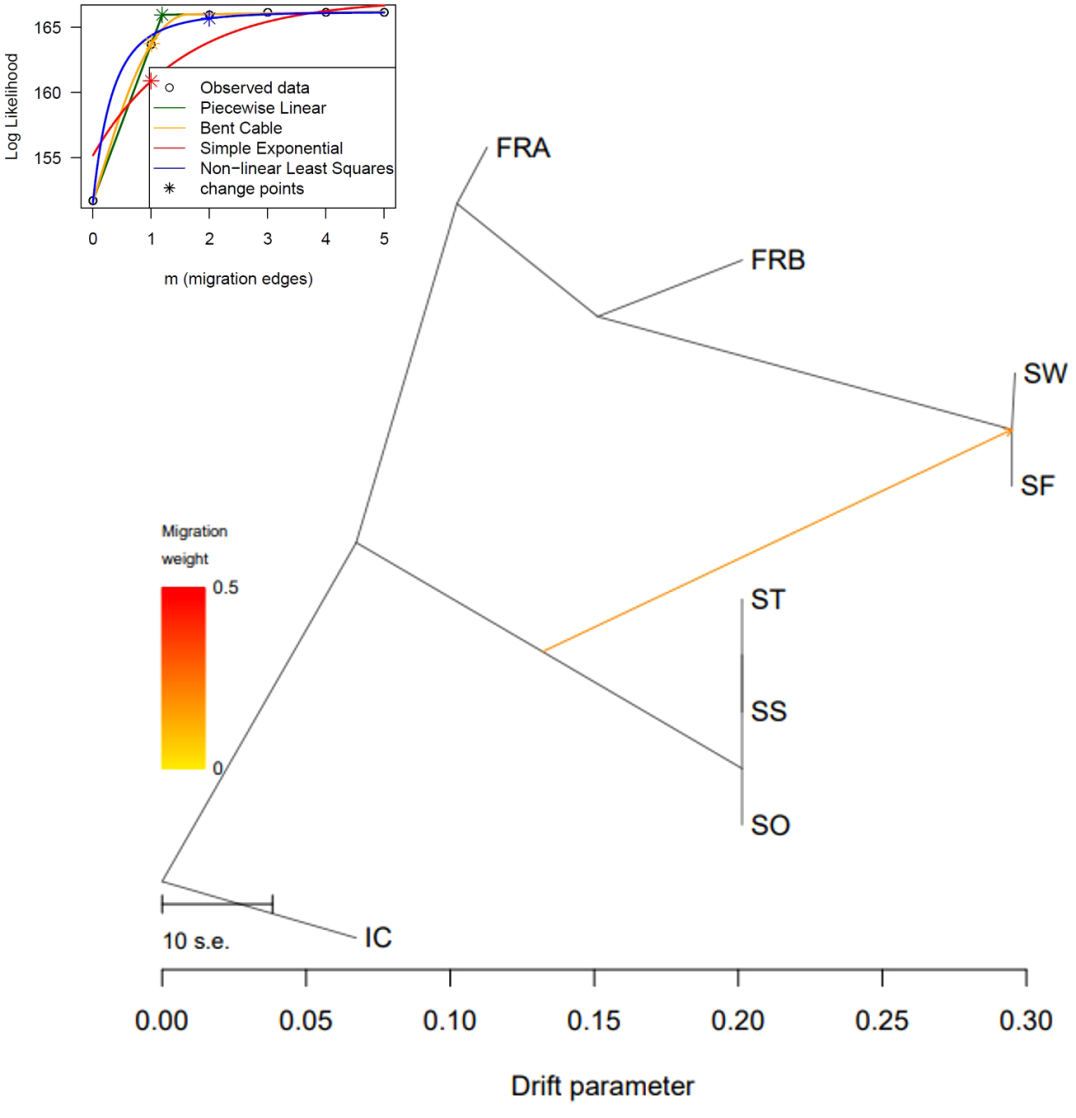
Evolutionary relationship of *Alliaria petiolata* samples inferred from TreeMix. Maximum- likelihood trees obtained allowing single gene flow explaining 99.9% of the variance. The color of the arrow indicates the gene flow weight, representing the fraction of ancestry derived from the gene flow edge.

We further explored potential reticulation among the 8 introduced and native garlic mustard populations via Neighbor-Net algorithm. Consistent with the two clustering results, four major clades were identified in the network tree (**Figure 6**). The three Samecheok populations (SS/SO/ST) and the SF/SW populations were closely related forming two major clades, whereas IC was distantly positioned from these two clusters (**Figure 6**). We found no clear reticulation signal among these three clusters except for a weak interconnection between the two clades (SF/SW and SO/SS/ST; **Figure 6**). However, genetic relationships among the genotypes of the two native populations were more complex showing intertwined relations. We also investigated potential gene flow among the 8 populations through TreeMix analysis. The overall topology of maximum likelihood (ML) tree inferred from TreeMix was congruent with the clustering patterns of Neighbor-Net and the two clustering analyses (**Figure 7**). Based on the long branch lengths among clades, strong drift effect can be presumed since each clade diverged from one another. Compared to no migration which explains 99.4% of the genetic covariance, adding one migration edge greatly improved the fit of the tree to the data (99.9% of the total covariance explained). On the population graph, we observed one migration (migration weight = ~ 0.2) from SO/SS/ST cluster to SF/SW cluster (**Figure 7**).

## 4 Discussion

Non-natives pass through a series of stages involving complex biotic and abiotic filters before becoming a successful invader (Theoharides and Dukes, 2007). While prevention is the ultimate strategy, early detection and immediate assessment of the colonization stages are crucial if initial screening fails, to prevent further spread (Simberloff et al., 2013). *Alliaria petiolata*, one of the most noxious alien plants in woodland habitats (Blossey et al., 2001), was first recorded in Korea just over ten years ago (Cho and Kim, 2012). Despite its high invasiveness reported elsewhere, the species has not received much attention in Korea due to its rather relaxed spread rate since the first emergence. Our study represents the first attempt to assess the colonization status and potential spread of *A. petiolata* in Korea using cytological and population-level genomic data. Our findings suggest that multiple introductions may have greatly influenced the garlic mustard’s invasion in Korea. In addition, based on our molecular data and the current distribution pattern, the currently observed slow spreading rate may be attributed to a potential lag phase.

Contrary to our initial hypothesis, we discovered that the cytological identity of *A. petiolata* introduced in Korea is rather complex. In our chromosome counting analysis, at least two chromosome numbers were observed (2n= 36 and 42; **Figure 2**). As the basic chromosome numbers reported for the species are X= 6 or 7, the examined samples appear to be hexaploids. Species with multiple genome copies (polyploids) likely benefit from heterosis and hybrid vigour, making them more successful colonizers than diploid non-natives (Te Beest et al., 2012; Suda et al., 2015; Mairal et al., 2023). Polyploidy may indeed be playing an important role during garlic mustard’s invasion in Korea. However, due to the limited sample numbers, our chromosome data should be interpreted with great caution. Future studies may further explore the cytological characteristics of the species with a larger sample size to draw meaningful conclusions regarding the ploidy of *A. petiolata* populations.

Surprisingly, measures of within-population genetic diversity observed in Korean populations were comparable to those of the two populations originating from the native region (**Table 1**). Given the inevitable founder effects during early stages of biological invasion, genetic diversity loss is commonly expected for a recently introduced species (Dlugosch and Parker, 2008; Dlugosch et al., 2015). Indeed, the six populations sampled from the introduced region (Korea) have experienced population bottlenecks although these populations are currently rebounding from the decline. However, we did not find significant reduction in Korean populations compared to the native populations (P < 0.05; **Table 1**). The result is somewhat consistent with a previous study, which found no pronounced population bottlenecks in introduced populations compared to native European populations (Durka et al., 2005). This suggests that the introduction of *A. petiolata* and its successful invasion may not necessarily accompany a bottleneck, contrary to general expectations. Alternatively, a bottleneck may have occurred during the introduction, but there could have been factors mitigating its impact, such as hybridization between divergent lineages introduced through multiple introductions (Dlugosch and Parker, 2008; Thompson et al., 2010). Therefore, we conducted a TreeMix analysis to investigate the potential influence of hybridization. The detection of only minor gene flow between two divergent lineages, originating from the three clusters, casts doubt on the potential effect of hybridization on the observed genetic diversity pattern (**Figure 7**).

Alloploidy may be an alternative explanation for the well-maintained genetic variation. As aforementioned, garlic mustards are predominantly hexaploids and primarily reproduce through selfing (Durka et al., 2005; Mullarkey et al., 2013). Indeed, the level of genetic variation was not notably high even in the native region, except for the Ho (**Table 1**). Interestingly, the Ho values were consistently high across all 8 populations investigated, with a mean Ho of 0.4. Given the species’ high selfing rate in nature, these elevated Ho values are even more noteworthy and deserving of attention. Although the exact cytotype remains unknown, based on the pattern of genetic diversity, the hexaploids examined in our study likely be allopolyploids resulting from past hybridization events. About 30% of the 1,172 loci used in downstream analyses were heterozygotic loci fixed for all genotypes included in the diversity analysis. The fixed heterozygosity, determined by codominant molecular markers, is considered evidence of allopolyploidy (Soltis and Soltis, 2000). Furthermore, allopolyploid origin of hexaploid *A. petiolata* was proposed based on the features of the reconstructed genome (Bayat et al., 2021). Accordingly, the unexpectedly high Ho resulting from a high rate of fixed heterozygosity in both native and introduced regions provides further evidence supporting the allopolyploid origin of hexaploid garlic mustard.

We found three genetic clusters within Korean populations through the two clustering analyses (**Figures 3**, **4**). The clustering patterns closely mirrored the topologies of population graph inferred from ML algorithm and the network tree (**Figures 5**, **6**). Despite this consistency, the genetic affinity among the clusters did not correspond to geographic proximity, as indicated by the Mantel test results (see **Figure 3**). A notable example of the incongruence was observed in Samchoek, where two clusters were assigned despite being within close geographic proximity (< 2 km). The significant discordance between geography and genetic similarity, as evidenced by the presence of two distinct clusters within a single city, suggests the possibility of multiple introductions during the initial stages of garlic mustard invasion. This hypothesis gains further support from the differing chromosome numbers found in each cluster. According to current cytological knowledge, the cluster represented by SF, with 42 chromosomes, likely originated from Central/Western Europe and/or North America, while the cluster containing SS population with 36 chromosomes likely originated from the Netherlands or Sweden (Frisch and Møller, 2012; Alabi et al., 2021). Alternatively, the four populations in Samcheok might have originated from the same source but with mixed chromosome numbers. However, because of the narrow distribution of *A. petiolata* with 36 chromosomes in its native range, this alternative hypothesis may be discounted.

In the meantime, the genetic similarity between the SF population in Samcheok and the SW population in Suwon implies that the founders of SW population might have been transported from SF population. IC population, on the other hand, markedly diverged from the remaining Korean populations based on both phylogenetic inferences, Neighbor-net and ML phylogenies, suggesting the introduction of a third source (**Figures 5**, **6**). Given that Incheon serves as a major port of entry to Korea, it is highly probable that IC population in Incheon, exhibiting distinct genetic characteristics, was introduced by this third source. This inference aligns with the plausible invasion scenario inferred by our data. Another interesting observation in the genetic divergence pattern among populations was the extremely low genetic differentiations among ST, SO, and SS populations, as well as between SF and SW populations. The low divergence may indicate the existence of continuous gene flow between these populations. However, seed dispersal of *A. petiolata* is spatially restricted, as revealed in a previous study (Biswas and Wagner, 2015), which limits gene flow among populations. Consequently, the combined effect of highly similar genotypes initially introduced into these populations and predominant selfing might have led to the extreme genetic similarity observed between these populations.

In North America, *A. petiolata* was estimated to expand across the landscape at a rate of 6,400 square kilometers per year, clearly demonstrating its noxious impact on the native plant community (Nuzzo, 1993; Rodgers et al., 2008). Despite its high invasive potential, the current stage of the *A. petiolata* invasion in Korea does not seem severe; the species is colonizing only a few restricted areas with a small number of populations (http://nature.go.kr/kbi/plant/ntrlz/selectNtpltDtl.do) within the past decade. The rather relaxed spread rate, despite the high invasive potential, suggests that the garlic mustard invasion in Korea may be at a “lag phase”. Coupled with a suit of biological features that serve as means to be a successful colonizer, polyploidy, predominantly found in *A. petiolata*, may promote the rate of success during Korean invasion. As suggested in Bayat et al. (2021) and further supported by our data, hexaploid accessions of garlic mustard are likely allohexaploids resulting from apparent hybridization events in the past. Past hybridization may also catalyze the evolution of invasiveness, contributing to garlic mustard’s invasion success (Ellstrand and Schierenbeck, 2000; Mesgaran et al., 2016; Gioria et al., 2023). Taken all together, our data highly suggest that the plant is likely to further spread upon reaching the breaking point of the lag phase, although the exact timing of the expansion cannot be determined. Accordingly, garlic mustard populations must be closely monitored to prevent it from becoming a noxious weed in Korea.

## Data availability statement

The original contributions presented in the study are publicly available. This data can be found here: National Center for Biotechnology Information (NCBI) BioProject database under accession number PRJNA1113695.

## Author contributions

T-YC: Writing – review & editing, Visualization, Investigation, Formal analysis, Data curation. DS: Writing – review & editing, Resources, Methodology, Funding acquisition, Conceptualization. AO: Writing – original draft, Validation. S-RL: Writing – review & editing, Writing – original draft, Visualization, Validation, Supervision, Project administration, Methodology, Investigation, Formal analysis, Conceptualization.
