# Supplementary material for: Unveiling a potential threat to forest ecosystems: molecular diagnosis of Alliaria petiolata, a newly introduced alien plant in Korea
Source: Front Plant Sci. 2024 Jul 1;15:1395676. doi: 10.3389/fpls.2024.1395676 (PMC11246967; doi:10.3389/fpls.2024.1395676)
Supplement: Supplementary file 1 [file DataSheet_1.docx]

Supplementary Material

**Unveiling a potential threat to forest ecosystems: molecular diagnosis of *Alliaria petiolata*, a newly introduced alien plant in Korea**

Tae-Young Choi^1^, Dong Chan Son^2^, Ami Oh^1^, Soo-Rang Lee^1*^

^1^ Department of Biology Education, College of Education, Chosun University, Gwangju, South Korea

^2^Division of Forest Biodiversity and Herbarium, Korea National Arboretum, Pocheon, Republic of Korea

*** Correspondence:**Soo-Rang Lee
ra1130@hotmail.com

# Supplementary Data

# Supplementary Information 1. Comprehensive parameters and commands employed for the ipyrad pipeline

import ipyrad.analysis as ipa

import ipyrad as ip

import ipyparallel as ipp

import pandas as pd

# ipcluster start -n 100 --cluster-id="ipyrad2" --daemonize

# After the cluster is running you can attach to it with ipyparallel

ipyclient = ipp.Client(cluster_id="ipyrad2")

data = ip.Assembly("France")

data.set_params("sorted_fastq_path", "./sample/*.fq.gz")

data.set_params("assembly_method", "reference")

data.set_params("reference_sequence", "./reference/GCA_020283515.1_Apet_EFCC3-3-20v4_genomic.fna")

data.set_params("datatype", "pairddrad")

data.set_params("restriction_overhang", "'CATG', 'AATT'")

data.set_params("clust_threshold", "0.9")

data.set_params("max_alleles_consens", "6")

data.set_params("max_Hs_consens", "0.1")

data.set_params("max_shared_Hs_locus", "1")

data.run("1234567", force=True, ipyclient=ipyclient)
